# Which outcome variables are associated with psychological inflexibility/flexibility for chronic pain patients? A three level meta-analysis

**DOI:** 10.3389/fpsyg.2022.1069748

**Published:** 2022-12-06

**Authors:** Shuanghu Fang, Dongyan Ding

**Affiliations:** School of Educational Science, Anhui Normal University, Wuhu, China

**Keywords:** meta-analysis, psychological flexibility, psychological inflexibility, chronic pain, psychological distress, pain intensity, acceptance commitment therapy (ACT)

## Abstract

The psychological flexibility model can be seen as a basis for an integrated and progressive psychological approach to chronic pain management. Some researchers suggest that psychological flexibility and inflexibility represent distinct processes and constructs. This meta-analysis is the first to provide a summary estimate of the overall effect size for the relationship between psychological (in)flexibility and common outcomes among chronic pain patients. The research protocol was registered in the International Prospective Register of Systematic Reviews (PROSPERO, https://www.crd.york.ac.uk/PROSPERO/), registration number CRD42021285705. Four databases were searched (PsycINFO; PubMed; Web of Science, CINAHL) along with reference lists. Thirty-six cross-sectional studies were included (7,779 participants). Meta-analyses (random effects model) indicated a significant medium negative association between psychological flexibility and pain intensity or functional impairment. The present study also indicated a significant small to medium association between psychological inflexibility and pain intensity, a nearly large association between psychological inflexibility and functional impairment as well as the quality of life, and a large association between psychological inflexibility and anxiety/depression. Due to the limited number of included studies, the relationship between risk behavior and psychological inflexibility may not be significant. Types of countries and instruments measuring psychological inflexibility may explain part of the heterogeneity. These findings may carry significant implications for chronic pain patients regarding the potential relationship between psychological inflexibility or flexibility and these outcomes. It may consequently form the basis for more robust testing of causal and manipulable relationships.

**Systematic review registration:**

https://www.crd.york.ac.uk/PROSPERO/, identifier: CRD42021285705.

## Introduction

Chronic pain is one of the most common health conditions worldwide in the general population (Brooks et al., 2017; Si et al., 2019; Rhodes et al., 2021) and is a source of distress and disability that affects all aspects of a patient's life. For example, chronic physical pain is linked to various psychological distress, such as anxiety, stress, and depression (Goldbart et al., 2020; Esteve et al., 2021). Furthermore, individuals in a state of psychological distress experience more intense pain, leading to a reciprocal reinforcement between psychological distress and pain (Davis et al., 2004; Goldbart et al., 2020). Chronic pain entails economic costs higher than other diseases, such as heart disease, diabetes, and cancer (Esteve et al., 2021). In addition to the monetary costs of treatment, other costs (e.g., disability compensation, loss of productivity, sleep problems, and increased healthcare utilization) are also common (Turk and Burwinkle, 2005). Therefore, it is imperative to develop an effective chronic pain management program for chronic pain patients.

There are a variety of treatments for chronic pain. However, medical and other previously traditional treatments are costly and of questionable effectiveness in treating persistent pain (Hoffman et al., 2007; Rhodes et al., 2021). Conversely, psychological treatments can benefit patients with chronic pain, specifically in terms of daily functioning and health-related quality of life (Hoffman et al., 2007; Rhodes et al., 2021). One of the noteworthy psychological treatments is acceptance and commitment therapy (ACT). The meta-analyses of ACT for chronic pain show that ACT was more effective than controls (i.e., no alternative intervention or treatment as usual) in terms of functioning, pain intensity, depression, and anxiety (Hughes et al., 2017; Trindade et al., 2021). ACT targets psychological flexibility, which is the primary determinant of mental health and effective action (Dionne et al., 2013). Psychological flexibility refers to an individual's ability to focus on the present moment, move toward their goals, and persist or change behaviors to serve valued ends. The opposite is psychological inflexibility which could be dysfunctional, ultimately contributing to and exacerbating psychopathology (Hayes et al., 2006; Daks et al., 2020; Fang and Ding, 2020). Psychological flexibility is established through six core ACT processes, i.e., acceptance, defusion, present moment, self-as-content, committed action, and values. In contrast, the opposite six dimensions constitute psychological inflexibility: lack of present moment awareness, lack of contact with values, inaction, self-as-content, fusion, and experiential avoidance (Hayes et al., 2006; Ding and Zheng, 2022).

The psychological flexibility model can be seen as a basis for an integrated and progressive psychological approach to chronic pain management (McCracken and Morley, 2014). This model fully integrates cognitive and environmental influences as the core processes of health and risk behaviors (McCracken and Morley, 2014). In chronic pain, psychological inflexibility is considered to be an important determinant of mood swings and ineffective living (Wicksell et al., 2008). It also leads to short-term relief behaviors, such as physical inactivity and avoidance of activities (i.e., remaining still or not going out of doors). However, these behaviors often prevent them from activities that increase their quality of life (e.g., visiting friends, working, or playing sports) (Maathz et al., 2020; Rhodes et al., 2021). Over time, the patient's strategy of avoiding negative psychological events produces a narrow and inflexible pattern of action, resulting in increased disability (Wicksell et al., 2008). In comparison, the flexibility model suggests that pain and suffering are inherent aspects of human life. Psychological flexibility, on the other hand, is characterized by accepting inner experiences, being mindful, and participating in actions aligned with individual goals and values, which may relieve the psychological burden and increase wellbeing (Maathz et al., 2020; Rhodes et al., 2021). Moreover, psychological flexibility is a malleable factor which is amenable to change through ACT (Hayes et al., 2012). It is known that outcome variables are usually organized into four categories, such as functioning (e.g., goal progress, pain interference, functional impairment, and subjective accounts of functioning), quality of life, mental health symptomology (e.g., symptoms of depression and/or anxiety), and observable behavioral changes (e.g., smoking cessation and risk medication-related behaviors) (Stockton et al., 2019). For the person with chronic pain, pain intensity has been the primary target for pain measurement (Rhodes et al., 2021). Therefore, understanding the relationship between psychological (in)flexibility and these outcomes for patients with chronic pain may have important implications for intervention and, thus, for organizations and patient care.

The previous study of patients with chronic pain has supported the role of the various components of psychological flexibility in their wellbeing and daily functioning (McCracken and Velleman, 2010), such as acceptance (Varallo et al., 2021, 2022), defusion, present moment, self-as-content (Nigol and Di Benedetto, 2020), committed action, and values (McCracken and Vowles, 2008). However, some studies suggest that global psychological flexibility may work better as a predictor of treatment outcome than measures of various components from the psychological flexibility model (Gilpin et al., 2019; Åkerblom et al., 2021). Besides, some meta-analytic results suggested that the individual dimensions of flexibility and inflexibility might represent distinct (yet related) processes and constructs (Rolffs et al., 2018; Daks and Rogge, 2020). Nonetheless, many studies have mixed the two, i.e., using instruments that measure inflexibility to measure flexibility (Dubey et al., 2020; Goldbart et al., 2020). Some researchers suggest that the acceptance and action questionnaire-II (AAQ-II), which has been widely used for measuring psychological flexibility, may be more appropriate for psychological inflexibility (Gilpin et al., 2019; Åkerblom et al., 2021). It is worth noting that different results also appeared for the same relationship between outcome variables, such as the relationship between psychological flexibility and pain severity. For example, one study (Allen et al., 2018) showed that the correlation coefficient of psychological flexibility and pain severity was not significant, while in another study, the effect size tended to be large (Vallejo et al., 2021).

As mentioned above, to date, no meta-analysis has summarized the relationship between psychological flexibility/inflexibility and these outcome variables in people with chronic pain. A meta-analysis of this subject is important to gain a more coherent understanding of these relationships, as the literature indicates chronic pain patients are already subject to intervention strategies designed to enhance psychological flexibility or reduce psychological inflexibility to improve their lives. Further analytical evidence is needed to examine whether and how psychological (in)flexibility could be associated with these outcomes for chronic pain patients, which would form the basis for more robust testing of causal and manipulable relationships.

Thus, the primary aim of this review was to identify and integrate all published findings on associations between psychological flexibility/inflexibility and these five outcomes, and address an analytic question about the magnitude and direction of the associations within chronic pain patients. A second aim is to determine which variables potentially moderate the relationship between psychological (in)flexibility and outcomes. We hypothesized that the following four moderators would systematically influence the effect: (1) the type of measurement, (2) the age of target sample, (3) the country, and (4) the proportion of females. A third research goal is to address descriptive questions about how these variables are being measured for chronic pain.

## Methods

### Selection of studies

The meta-analysis was reported following the Preferred Reporting Items for Systematic Reviews and Meta-analyses (PRISMA) statement (Page et al., 2021). The research protocol was registered in the International Prospective Register of Systematic Reviews (PROSPERO, https://www.crd.york.ac.uk/PROSPERO/) to minimize the risk of bias in this systematic review, registration number CRD42021285705.

We searched PsycINFO, PubMed, Web of Science, and CINAHL, all of which were done on October 1, 2022. No date restrictions were applied to the search to maximize the search strategy. The main search terms used included keywords and free words: (“experiential avoidance” OR “psychological acceptance” OR “psychological flexibility” OR “psychological inflexibility”) AND (“chronic pain” OR cancer OR fibromyalgia). In addition, reference lists of eligible studies, relevant review articles, and relevant meta-analyses were manually searched to minimize potential publication bias.

### Inclusion criteria

(a) The sample population included chronic pain patients, included cancer patients, and fibromyalgia patients;
(b) Psychological flexibility or psychological inflexibility was measured.
(c) The relationship between psychological (in)flexibility and outcome measures (i.e., pain severity/intensity, functioning, quality of life/wellbeing, mental health symptomology, and behavioral changes) were reported with Pearson's *r* correlation coefficient or standardized regression coefficients (β).

If studies reported standardized regression coefficients, they would be converted into correlation coefficients using the equation: *r* = β + *0.05*λ (λ = 1 if β is positive and λ = 0 if β is negative) (Liu et al., 2021).

### Exclusion criteria

(a) Review, meta-analysis, or theoretical articles;
(b) Without reporting Pearson's *r* correlation coefficient or standardized regression coefficients.

Difficulties in deciding the selection were discussed between the two authors. Any ambiguity about studying eligibility was settled *via* discussion. A full consensus was reached between the two authors according to the criteria.

### Data extraction and coding

We completed the data extraction and inspection together. If there were disagreements, agreements would be reached through a full consultation. Extracted data include: authors and year of publication, country, instruments, study characteristics (e.g., sample size, mean age, percentage of female), and effect sizes.

We created dummy variables for countries and tools of outcome measures (i.e., psychological flexibility or inflexibility, psychological distress, dysfunction, pain intensity, behavioral outcomes, and quality of life). We divided countries into Eastern and Western according to their geographical distribution. If the measurement was used only in one effect size, it would be coded as “other” to reduce the number of dummy variables (Ding and Zheng, 2022). The dummy variables were mutually exclusive.

### Data analysis

There is a discrepancy between the instruments measuring psychological flexibility and the constructs the authors claim to be measured. For example, AAQ-II (Bond et al., 2011) is more appropriate for measuring psychological inflexibility (Kashdan et al., 2020), while some studies use this to measure alternative constructs such as “psychological acceptance” or “experiential avoidance.” As such, the AAQ-II was considered a measure of psychological inflexibility in the present study. The directions of their effects were adjusted accordingly regardless of the construct defined within the individual study. Other constructs were categorized according to the author's classification. For example, if the authors use The Brief Pain Response Inventory (BPRI) to measure psychological flexibility, this study classified it as psychological flexibility.

If studies reported a range of outcome measures, the authors selected the outcome measure related to pain severity/intensity, functioning, quality of life/wellbeing, psychological distress, negative effects, positive effects, and behavioral outcomes. All relevant effect sizes would be extracted for the same category because studies report multiple effect sizes due to several relevant outcome measures and/or subscales of overarching concepts, e.g., “physical disability” and “psychosocial disability” as subscales of functioning (Jurkovich et al., 1995).

Due to differences in measurement tools, the effect sizes were analyzed using the random effects model in R version 4.1.2 (R Core Team, 2016) with the packages metafor and meta (Viechtbauer, 2010). A three-level meta-analytic model was used to synthesize effect sizes and conduct moderator analyses (Cheung, 2014). This model can explore three sources of variance: sampling variance of the observed effect sizes (Level 1), the variance between effect sizes from the same study (Level 2), and variance between studies (Cheung, 2014; Ding and Zheng, 2022). The significant variance of level 2 and level 3 indicated a heterogeneous effect size distribution. In addition, heterogeneity was also tested using the 75% rule (Assink and Carlijn, 2016), which contends that heterogeneity can be considered substantial if <75% of the overall variance can be attributed to level 1. Therefore, potential moderating effects would be examined according to the significant variance of level 2 or level 3 and the 75% rule. We first assess the (potential) moderating effects separately in the univariate model. If the effects were significant, we extended the meta-analytic model by adding all significant moderating variables simultaneously (Assink and Carlijn, 2016). Additionally, if there was no significant difference between the three-level model and the two-level model, then the results of the two-level model would be used according to *Occam's razor* (Harrer et al., 2021). The heterogeneity among the results of the two-level model would be tested by the *Q* test and the *I*^2^ test (Higgins et al., 2022). If *I*^2^ > 50%, it is suggested to have moderate-to-high heterogeneity. All model parameters were estimated using the restricted maximum likelihood method.

All extracted effects were converted to Fisher's Z values and weighted by sample size before analysis. These effects were then meta-analyzed, and the results were subsequently converted back to correlations for interpretation (Lipsey and Wilson, 2001). Following Cohen's convention, the magnitude of effect for *r* is classified as small (0.10), medium (0.30), or large (0.50) with 95% CI. Outliers of the effect size were inspected and removed. Outliers were defined as deviations greater than three standard deviations from the mean (Chiang et al., 2022).

Publication bias was first tested by Egger's regression test for funnel plot asymmetry (Kim et al., 2021; Higgins et al., 2022). We included sampling variance as a moderator and re-estimated the three-level model, and the significant effect of sampling variance was considered as an indicator of publication bias (Kim et al., 2021). Besides, a fail-safe N analysis was conducted to estimate the number of missing studies that would be needed to reduce the overall effect size insignificantly. If N was larger than 5k+10 (k was the number of included relevant studies in the present meta-analysis), then publication bias would be suggested as minimal. If publication bias were to be found, *trim and fill* analyses were performed to provide an adjusted average effect size (Duval and Tweedie, 2000) to correct. Sensitivity analyses were also conducted to test whether estimates were robust.

## Results

### Description of studies

#### Studies characteristics

**Figure 1:**
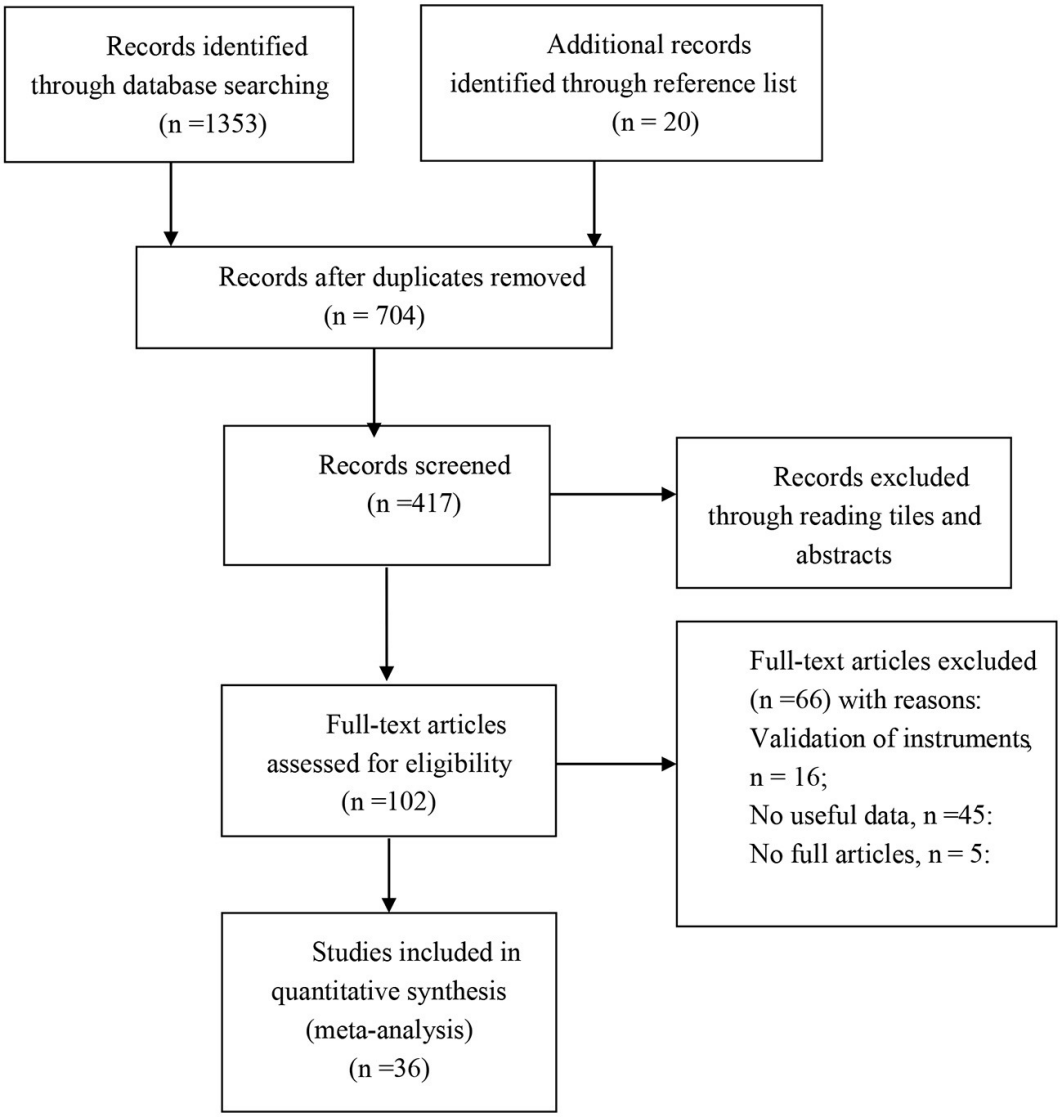
Study flow diagram.

Initially, 1,373 citations were identified by searching electronic bibliographic databases and reference lists. After detecting duplicates and screening titles and abstracts for relevance, 102 articles were identified as being potentially eligible for further assessment. After reading the full text of each article, 36 studies met the criteria and were included (see Figure 1 for the details).

The characteristics of the 36 included studies are summarized in Table 1. Sample sizes for included studies ranged from 34 to 1,522 (total participants = 7,779). The mean age of participants in these studies ranged from 18.86 to 73.8. The proportion of females ranged from 0 to 100%.

**Table 1:** Study characteristics and key findings.

**References**	**Country**	**PF/PI**	**PF/PImeasures**	**Outcomes**	**Outcome measures**	***r***	***M*** **age**	**%female**	***n***
Allen et al. (2018)	USA	Inflexibility	PIPS	Pain intensity	Pain intensity	−0.05	23.9	59	40
		Inflexibility	PIPS	Dysfunction	PROMIS-PIS	0.54	23.9		
Barke et al. (2015)	Germany	Inflexibility	PIPS-avoidance	Dysfunction	PDI	0.46	51	70.3	182
		Inflexibility	PIPS-avoidance	Dysfunction	QBPDS	0.42	51		
		Inflexibility	PIPS-fusion	Dysfunction	PDI	0.31	51		
		Inflexibility	PIPS-fusion	Dysfunction	QBPDS	0.14	51		
		Inflexibility	PIPS-avoidance	Anxiety	HADS	0.37	51		
		Inflexibility	PIPS-fusion	Anxiety	HADS	0.22	51		
		Inflexibility	PIPS-avoidance	Depression	HADS	0.48	51		
		Inflexibility	PIPS-fusion	Depression	HADS	0.12	51		
Berrocal et al. (2016)	Italy	Inflexibility	AAQ-II	Anxiety	HADS-Anxiety	0.61	47.62	100	42
		Inflexibility	AAQ-II	Depression	HADS-Depression	0.55	47.62		
Brown et al. (2020)	USA	Inflexibility	PIPS-avoidance	Pain intensity	BPI-severity	0.32	60.15	44.3	61
		Inflexibility	PIPS-avoidance	Dysfunction	BPI-functional impairment	0.54	60.15		
		Inflexibility	PIPS-avoidance	Psychological distress	NCCN-DT	0.33	60.15		
		Inflexibility	PIPS-fusion	Pain intensity	BPI-severity	0.39	60.15		
		Inflexibility	PIPS-fusion	Dysfunction	BPI-functional impairment	0.38	60.15		
		Inflexibility	PIPS-fusion	Psychological distress	NCCN-DT	0.36	60.15		
Carvalho et al. (2020)	Portugal	Inflexibility	PIPS-avoidance	Pain intensity	NPRS	0.25	50.49	100	49
		Inflexibility	PIPS-avoidance	Depression	DASS-21	0.61	50.49		
		Inflexibility	PIPS-avoidance	Dysfunction	PDI	0.41	50.49		
		Inflexibility	PIPS-fusion	Pain intensity	NPRS	0.42	50.49		
		Inflexibility	PIPS-fusion	Depression	DASS-21	0.45	50.49		
		Inflexibility	PIPS-fusion	Dysfunction	PDI	0.28	50.49		
Cary et al. (2017)	United States or Canada	Flexibility	BPRI	Pain intensity	Pain intensity	−0.29	49.75	85	136
		Flexibility	BPRI	Anxiety	PASS-20	−0.57	49.75		
		Flexibility	BPRI	Dysfunction	Physical activity	−0.27	49.75		
Dudek et al. (2016)	USA, UK, Australia	Inflexibility	AAQ-II	Quality of life	SWLS	−0.28	35–54	100	120
		Inflexibility	AAQ-II	Quality of life	WHOQOL-BREF	−0.48	35–55		
		Inflexibility	AAQ-II	Pain intensity	Symptom severity	0.06	35–56		
Fish et al. (2013)	USA (30.1%), Ireland (30.1%), England (21.4%)	Inflexibility	AAQ-II	Depression	HADS-Depression	0.63	44.07	79.54	535
		Inflexibility	AAQ-II	Anxiety	HADS-anxiety	0.71	45.07		
		Inflexibility	AAQ-II	Dysfunction	BPI-functional impairment	0.38	47.07		
		Inflexibility	AAQ-II	Pain intensity	BPI	0.19	47.07		
		Inflexibility	PIPS	Depression	HADS-Depression	0.66	44.07		
		Inflexibility	PIPS	Anxiety	HADS-anxiety	0.5	45.07		
		Inflexibility	PIPS	Dysfunction	BPI-functional impairment	0.52	47.07		
		Inflexibility	PIPS	Pain intensity	BPI	0.28	47.07		
Gandolfi et al. (2021)	Italy	Inflexibility	AAQ-II	Quality of life	Stroke Impact Scale	−0.48	63.96	32	50
Gentili et al. (2019)	Sweden	Inflexibility	PIPS	Pain intensity	Pain intensity	0.27	47.4	81	252
		Inflexibility	PIPS	Anxiety	GAD-7	0.34	47.4		
		Inflexibility	PIPS	Dysfunction	Pain interference index	0.67	47.4		
		Inflexibility	PIPS	Depression	PHQ-9	0.51	47.4		
		Inflexibility	PIPS	Bad behavior	Opioid use	0.12	47.4		
Goldbart et al. (2020)	Israel	Inflexibility	AAQ-II	Positive Affects	SPANE	0.11	58.3	57.1	177
González-Fernández et al. (2017)	Spain	Inflexibility	AAQ-II	Anxiety	HADS-Anxiety	0.77	52.4	100	122
		Inflexibility	AAQ-II	Depression	HADS-Depression	0.79	52.4		
		Inflexibility	AAQ-II	Psychological distress	Brief Symptom Inventory 18	0.7	52.4		
		Inflexibility	AAQ-II	Quality of life	EORTC QLQ-C30	−0.5	52.4		
		Inflexibility	AAQ-II	Pain intensity	EORTC QLQ-C30	0.31	52.4		
Hulbert-Williams and Storey (2016)	UK	Inflexibility	AAQ-II	Anxiety	HADS-Anxiety	0.6	61.4	55	129
		Inflexibility	AAQ-II	Depression	HADS-Depression	0.47			
		Inflexibility	AAQ-II	Quality of life	FACT-G	−0.47			
Kato et al. (2021)	Japan	Inflexibility	AAQ-II	Depression	PHQ-9	0.53	18.86	100	473
Koppert et al. (2021)	The Netherlands	Flexibility	Flexibility Index Test-60 (FIT-60)	Pain intensity	RANDSF-36	−0.53	47.7	80	1,522
Kwok et al. (2016)	China	Inflexibility	AAQ-II	Dysfunction	BPI-interference	0.38	Adults	67	100
		Inflexibility	AAQ-II	Pain intensity	BPI-severity	0.18			
		Inflexibility	AAQ-II	Psychological distress	HADS	0.69			
Lewson et al. (2021)	USA	Inflexibility	AAQ-II	Pain intensity	PROMIS	0.28	63.16	52.22	203
		Inflexibility	AAQ-II	Anxiety	PROMIS	0.66			
		Inflexibility	AAQ-II	Depression	PROMIS	0.67			
Lv et al. (2021)	China	Inflexibility	AAQ-II	Depression	SDS	0.31	37.9	43.9	82
		Inflexibility	AAQ-II	Anxiety	SAS	0.29			
		Inflexibility	AAQ-II	Quality of life	FACT-G	−0.34			
Maathz et al. (2020)	Sweden	Inflexibility	PIPS	Pain intensity	The Pain subscale of the FSFI	0.25	22.7	100	130
Marcelino et al. (2022)	Portugal	Inflexibility	PIPS	Pain intensity	DC/TMD	0.52	44.88	79.4	34
		Inflexibility	PIPS	Pain intensity	ICDH 3	0.39	45.92	65.6	61
McCracken and Zhao-O'Brien (2010)	UK	Inflexibility	AAQ-II	Depression	BC-MDI	0.69	42.4	63.9	144
		Inflexibility	AAQ-II	Anxiety	PASS-20	0.59			
		Inflexibility	AAQ-II	Dysfunction	SIP-psychosoc disability	0.65			
		Inflexibility	AAQ-II	Dysfunction	SIP-physical disability	0.42			
		Inflexibility	AAQ-II	Depression	BC-MDI	0.69			
		Inflexibility	AAQ-II	Dysfunction	SIP-psychosoc disability	0.65			
		Inflexibility	AAQ-II	Dysfunction	SIP-physical disability	0.42			
McCracken and Jones (2012)	UK	Inflexibility	AAQ-II	Depression	BCMDI	0.65	64.3	62.5	40
		Inflexibility	AAQ-II	Anxiety	PASS-20	0.67			
		Inflexibility	AAQ-II	Dysfunction	SIP-physical disability	0.45			
		Inflexibility	AAQ-II	Dysfunction	SIP-psychological disability	0.64			
		Inflexibility	AAQ-II	Pain intensity	Average pain intensity	0.27			
McCracken and Velleman (2010)	UK	Flexibility	AAQ	Pain intensity	pain intensity	−0.28	61.5	58.2	239
		Flexibility	AAQ	Dysfunction	SF-36-physical functioning	−0.27			
		Flexibility	AAQ	Dysfunction	SF-36-social functioning	−0.52			
		Flexibility	AAQ	Dysfunction	SF-36-emotional functioning	−0.6			
McCracken et al. (2011)	UK	Flexibility	AAQ	Dysfunction	SIP	−0.28	43.8	63	159
		Flexibility	AAQ	Pain intensity	PVAS	−0.13			
McCracken et al. (2013)	UK	Inflexibility	AAQ-II	Depression	BCMDI	0.71	43	69.3	150
		Inflexibility	AAQ-II	Pain intensity		0.11			
		Inflexibility	AAQ-II	Anxiety	PASS-20	0.62			
		Inflexibility	AAQ-II	Dysfunction	SIP-physical disability	0.1			
		Inflexibility	AAQ-II	Dysfunction	SIP-psychosoc disability	0.6			
Nagasawa et al. (2022)	Japan	Inflexibility	PIPS-avoidance	Dysfunction	PDAS	0.56	73.8	74	145
		Inflexibility	PIPS-avoidance	Pain intensity	PVAS	0.25			
		Inflexibility	PIPS-fusion	Dysfunction	PDAS	0.36			
		Inflexibility	PIPS-fusion	Pain intensity	PVAS	0.03			
Novakov (2021)	Serbia	Inflexibility	AAQ-II	Depression	DASS-21 (Depression)	0.57	58.36	100	64
		Inflexibility	AAQ-II	Anxiety	DASS-21 (Anxiety)	0.49			
		Inflexibility	AAQ-II	Dysfunction	UEFI	0.48			
		Inflexibility	AAQ-II	Quality of life	QOL-BC	−0.56			
Rhodes et al. (2021)	USA	Inflexibility	AAQ-II	Pain intensity	BPI-severity	0.44	57	32.3	99
		Inflexibility	AAQ-II	Dysfunction	BPI-functional impairment	0.58			
		Inflexibility	AAQ-II	Bad behavior	SOAPP-R	0.63			
Scott et al. (2017)	UK	Inflexibility	AAQ-II	Pain intensity	Pain Intensity	0.23	69.3	61.7	60
		Inflexibility	AAQ-II	Dysfunction	The SF-36 (physical functioning)	0.32			
		Inflexibility	AAQ-II	Dysfunction	The SF-36 (social functioning)	0.41			
		Inflexibility	AAQ-II	Depression	PHQ-9	0.51			
Sevier-Guy et al. (2021)	UK	Flexibility	CompACT-18	Quality of life	POPUS	0.37	68.5	0	144
		Flexibility	CompACT-18	Psychological distress	DASS-21	−0.67			
Trompetter et al. (2014)	The Netherlands	Inflexibility	PIPS	Dysfunction	MPI	0.46	43.7	72.2	428
		Inflexibility	PIPS	Quality of life	Rand-36 Health Survey	−0.54			
		Inflexibility	PIPS	Dysfunction	Rand-36 Health Survey	0.26			
		Inflexibility	PIPS	Dysfunction	PDI	0.39			
		Inflexibility	PIPS	Pain intensity	NRS	0.25			
Vallejo et al. (2021)	Spain	Inflexibility	AAQ-II	Pain intensity	CISF	0.72	51.63	94.7	187
		Inflexibility	AAQ-II	Dysfunction	FIQ	0.6			
Wicksell et al. (2008)	Sweden	Inflexibility	PIPS	Dysfunction	MPI-S	0.77	45.5	80.8	203
		Inflexibility	PIPS	Pain intensity	MPI-S	0.35			
		Inflexibility	PIPS	Quality of life	SF-12	−0.39			
		Inflexibility	PIPS	Quality of life	SF-12	−0.38			
		Inflexibility	PIPS	Quality of life	Perceived quality of life	−0.63			
Wicksell et al. (2010)	Sweden	Inflexibility	PIPS	Quality of life	SWLS	−0.51	49	74.8	611
		Inflexibility	PIPS	Pain intensity		0.3			
		Inflexibility	PIPS	Dysfunction	PDI	0.53			
		Inflexibility	PIPS	Anxiety	HAD	0.51			
		Inflexibility	PIPS	Depression	HAD	0.58			
Yang et al. (2017)	Singapore	Inflexibility	AAQ-II	Pain intensity	Pain intensity	0.18	45.27	56	200
		Inflexibility	AAQ-II	Dysfunction	BPI	0.38			
		Inflexibility	AAQ-II	Depression	PHQ-9	0.52			
		Inflexibility	AAQ-II	Depression	PHQ-9	0.37			
Zvolensky et al. (2022)	USA	Inflexibility	PIPS	Anxiety	ASI-III	0.64	40.26	87.2	406
		Inflexibility	PIPS	Pain intensity	GCPS	0.35			
		Inflexibility	PIPS	Dysfunction	Sheehan disability scale	0.41			

AAQ, Acceptance and Action Questionnaire; PIPS, Psychological Inflexibility in Pain Scale; BPRI, The Brief Pain Response Inventory; PPFQ, Parent Psychological Flexibility Questionnaire; CompACT, Comprehensive Assessment of Acceptance and Commitment Therapy Processes; ASI-III, Anxiety Sensitivity Index-III; BCMDI, British Columbia Major Depression Inventory; HADS, The Hospital Anxiety and Depression Scale; PHQ, Patient Health Questionnaire; DASS, Depression Anxiety Stress Scales; PNAS, Positive and Negative Affect Scale; PASS, Pain Anxiety Symptoms Scale; NCCN-DT, National Comprehensive Cancer Network Distress Thermometer; HAD, Hospital Anxiety and Depression Scale; GAD-7, Generalized Anxiety Disorder-7; PROMIS, Patient-Reported Outcomes Measurement Information System; SDS, Self-rating Depression Scale; SAS, Self-rating Anxiety Scale; NPRS, Numeric Pain Rating Scale; NRS, Numeric Rating Scale; CISF, Combined Index of Severity of Fibromyalgia; RANDSF-36, Short Form Health Survey; DC/TMD, Diagnostic Criteria for Temporomandibular Disorders; ICDH 3, International Classification of Headache 3; PVAS, Pain visual analog scale; QOL-BC, Quality of Life Instrument-Breast Cancer Patient Version; PedsQL-SF15, Pediatric QOL Inventory Generic Core Scales Short Form; WHOQOL-BREF, World Health Organization Quality of Life scale; SPANE, Scale of Positive and Negative Experience; SF-12, Short Form-12 Health Survey; SWLS, Satisfaction With Life Scale; FACT-G, Functional Assessment of Cancer Therapy—General; POPUS, Patient Orientated Prostate Utility Scale; SIP, Sickness Impact Profile; BPI, Brief Pain Inventory; PDI, Pain Disability Index; GCPS, Graded Chronic Pain Scale; FACT-G, Functional Assessment of Cancer Therapy—General; PROMIS-PIS, PROMIS Pain Interference scale; QBPDS, Quebec Back Pain Disability Scale; MPI-S, Multidimensional Pain Inventory-Swedish version; PDI, Pain Disability Index; FIQ, Fibromyalgia Impact Questionnaire; UEFI, Upper Extremity Functional Index; PDAS, Pain Disability Assessment Scale; SOAPP-R, Screener and Opioid Assessment for Patients with Pain—Revised.

#### The measurements

Thirteen studies used the Psychological Inflexibility in Pain Scale (PIPS) to measure psychological inflexibility, and AAQ-II was used in 19 studies. Psychological flexibility was measured with AAQ (two studies), The Brief Pain Response Inventory (BPRI) (one study), the Comprehensive Assessment of Acceptance and Commitment Therapy Processes (CompACT-18) (one study), and Flexibility Index Test-60 (FIT-60).

Anxiety was measured with Hospital Anxiety and Depression Scale (HADS)-Anxiety (eight studies), Pain Anxiety Symptoms Scale (PASS)-20 (four studies), Generalized Anxiety Disorder-7 (one study), Self-rating Anxiety Scale (one study), and Depression Anxiety Stress Scale (DASS-21) (one study), Anxiety Sensitivity Index-III (ASI-III) (one study), Patient-Reported Outcomes Measurement Information System (PROMIS) (one study). Depression was measured with HADS-Depression (eight studies), Patient Health Questionnaire (PHQ)-9 (four studies), BCMDI (three studies), DASS-21-Depression (three studies), PROMIS (one study), and Self-rating Depression Scale (one studies). Functional impairment was mostly measured with Brief Pain Inventory-functional impairment subscale (BPI) (five studies), and Sickness Impact Profile (SIP) (four studies), Pain Disability Index (PDI) (four studies). Quality of life was measured with the Satisfaction with Life Scale (SWLS), Short Form-12 Health Survey (SF-12), World Health Organization Quality of Life scale 3 (WHOQOL-BREF), and Stroke Impact Scale (SIS). More details can be seen in Table 1.

### Meta-analyses

#### Psychological inflexibility/flexibility and pain intensity

**Figure 2:**
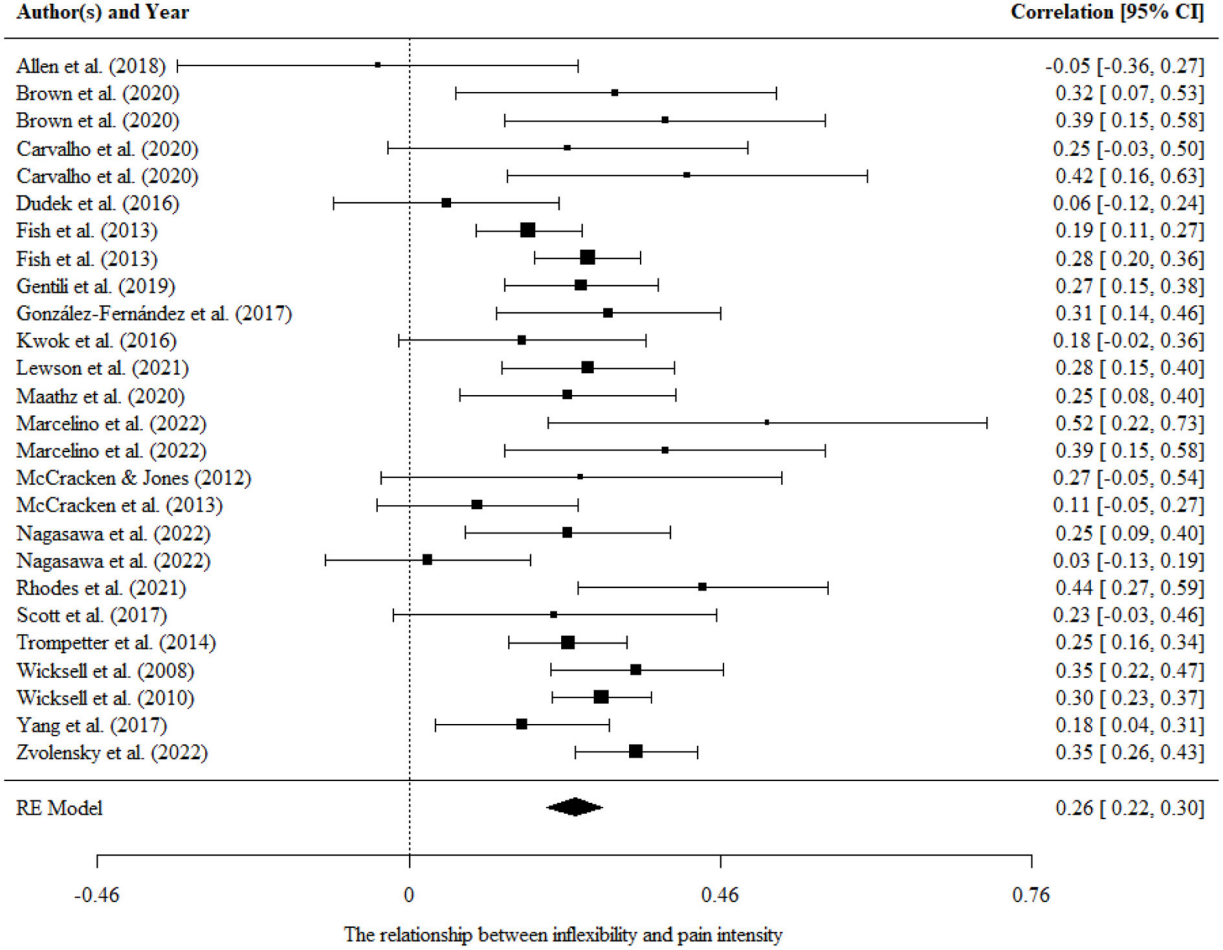
Forest plot for the relationship between psychological inflexibility and pain intensity.

There was no significant difference between the two-level and three-level models, so we used the results of the two-level model according to *Occam's razor* (Harrer et al., 2021). Aggregating across 26 correlational studies that examined the relationship between psychological inflexibility and pain intensity, the overall effect size was statistically significant and nearly medium (*r* = 0.26, 95% CI = 0.22, 0.30, *p* < 0.001, *n* = 4,839, *I*^2^ = 43.7%; *Q* = 44.37, *df* = 25, *p* < 0.001). The results were presented in a forest plot in Figure 2.

Moderating effects of mean age, percentage of females, country, and measurement tools were evaluated separately in univariate models. We found a significant moderating effect of the country and measurement of psychological inflexibility on the association in univariate models, as shown by the results of the omnibus test [*F*_(1, 24)_ =5.22, *p* < 0.05; *F*_(1, 24)_ =4.65, *p* < 0.05]. When the two significant moderating variables were added simultaneously, only the regression coefficient of the eastern country (−0.11) was significant [*t*_(96)_ = −2.20, *p* < 0.005], and the test for residual heterogeneity was not significant (*Q* = 32.33, *df* = 24, *p* > 0.05). It was suggested that the relationship between psychological inflexibility and pain intensity is larger in the western country than in the eastern country.

There was no publication bias in the Egger tests (*p* > 0.1) and fail-safe N analysis (*N* = 3,515) on the relationship between psychological inflexibility and pain intensity, and further sensitivity analysis showed that the results were robust.

Four correlational studies examined the relationship between psychological flexibility and pain intensity. The overall effect size was statistically significant and medium (*r* = −0.32, 95% CI = −0.49, −0.14, *p* < 0.001, *I*^2^ = 91.21%; *Q* = 49.65, *df* = 3, *p* < 0.001). The *I*^2^ and *Q* statistic indicated high variation between studies attributable to heterogeneity.

#### Psychological inflexibility/flexibility and functional impairment

**Figure 3:**
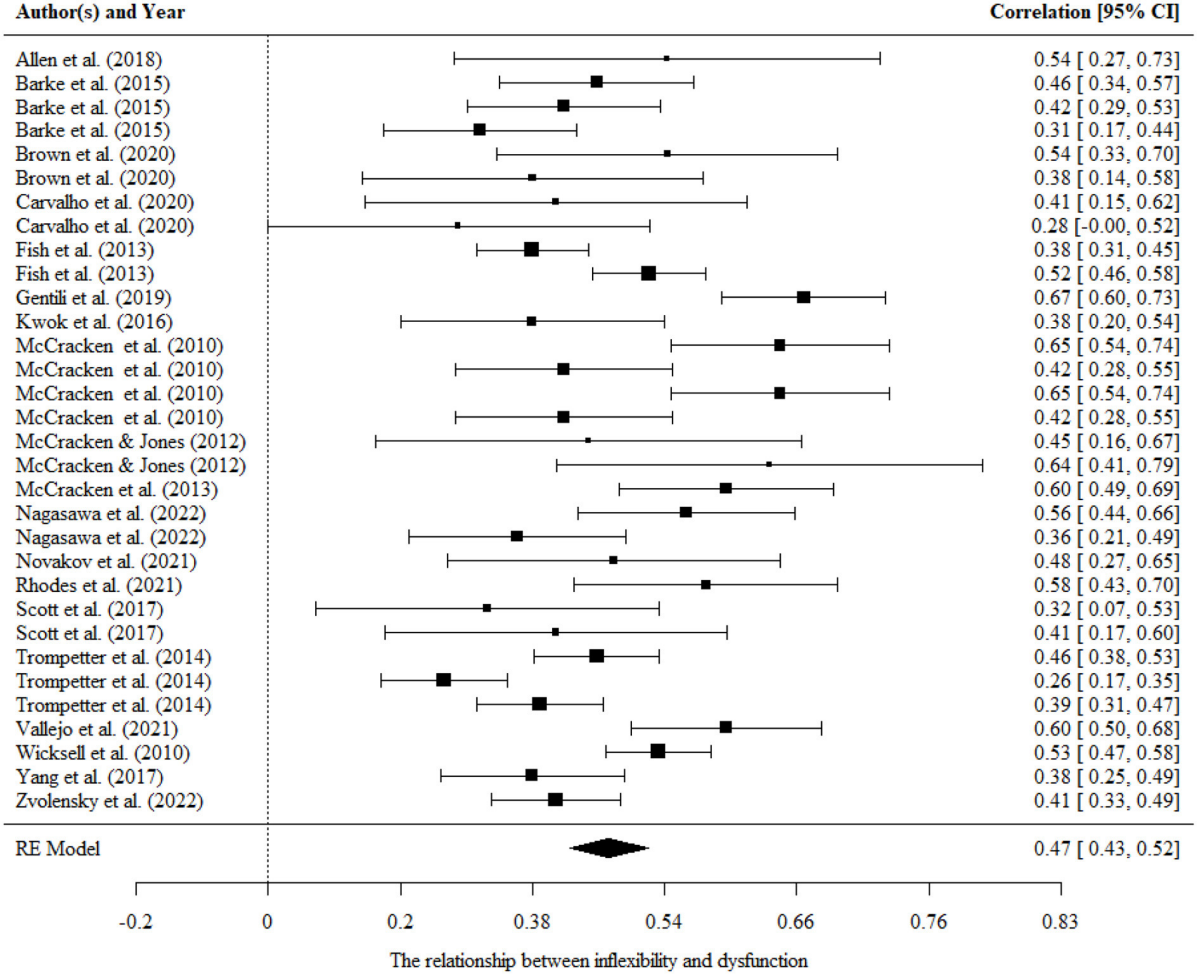
Forest plot for the relationship between psychological inflexibility and functional impairment.

The results indicated a nearly large pooled effect size estimate (*r* = 0.49, 95% CI = 0.43, 0.52, *p* < 0.001) for the relationship between psychological inflexibility and functional impairment. The results are presented in Figure 3, and more details can be seen in Table 2. There were significant variances within the same studies (i.e., level 2 variance). However, no significant moderating variables were found in this study. That is, mean age, country, and measurements were not potential moderating variables. Possible reasons were summarized in the Discussion section.

**Table 2:** The relationship between psychological (in)flexibility and outcomes, and heterogeneity.

		**# Studies**	**# ES**	**Mean r**	**95% CI**	**% var. at level 1**	**Level 2 variance**	**% Var. at level 2**	**Level 3 variance**	**% Var. at level 3**	**Heterogeneity**		
											**Q**	***P***	***I^2^*** **(%)**
PI	Pain intensity	21	26	0.26	0.22, 0.30	56.09	0.00	27.39	0.00	16.52	44.37	<0.01	43.70
	Dysfunction	19	32	0.48	0.43, 0.52	24.57	0.01^**^	62.23	0.00	13.21			
	Anxiety	12	14	0.55	0.47, 0.62	14.50	0.16^**^	85.50	0.00	0.00			
	Depression	16	20	0.57	0.51, 0.62	23.52	0.00	3.79	0.02^*^	72.69			
	Quality of life	9	13	−0.47	−0.53, −0.41	36.24	0.1^**^	63.75	0.00	0.00			
	Risk behaviors	2	2	0.41	−0.18, 0.77						27.10	<0.01	93.60
PF	Pain intensity	4	4	−0.32	−0.49, −0.14						49.65	<0.01	91.21
	Dysfunction	3	5	−0.40	−0.60, −0.17	12.60	0.19^**^	84.63	0.03	2.77			

PI, psychological inflexibility; PF, psychological flexibility; # Studies, number of studies; # ES, number of effect sizes; CI, confidence interval; Sig, significance; Mean r, Mean effect size expressed as a Pearson's correlation; Var, variance; Level 1 variance = sampling variance of observed effect sizes; Level 2 variance = variance between effect sizes extracted from the same study; Level 3 variance = variance between studies.

* p < 0.05;

** p < 0.01.

There was no publication bias in the Egger tests (*p* > 0.1) and fail-safe N analysis (*N* = 16,016) on the relationship between psychological inflexibility and dysfunction, and further sensitivity analysis showed that the results were robust.

Three studies, including five correlations, examined the relationship between psychological flexibility and functional impairment. The overall effect size was statistically significant and medium (*r* = −0.40, 95% CI = −0.60, −0.17, *p* < 0.001). There were significant variances within level 2. Fail-safe N analysis (*N* = 238) suggested that there was no publication bias, and sensitivity analysis showed that the results were robust.

#### Psychological inflexibility/flexibility and anxiety

**Figure 4:**
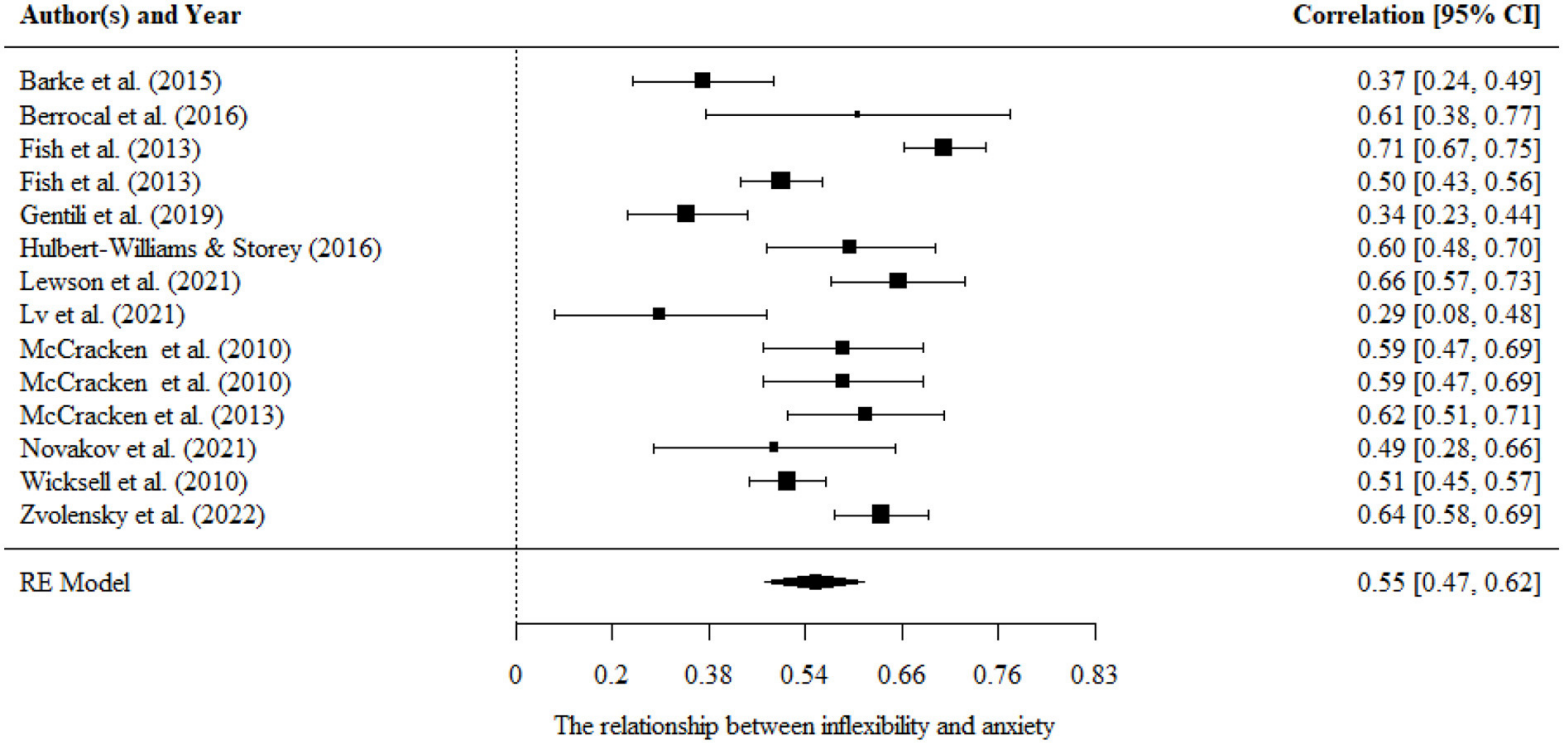
Forest plot for the relationship between psychological inflexibility and anxiety.

Aggregating across 14 correlations in 12 studies that examined the relationship between psychological inflexibility and anxiety, the overall effect size was statistically significant and large (*r* = 0.55, 95% CI = 0.43, 0.52, *p* < 0.001). The results are presented in Figure 4.

There were significant variances within level 2. Moderating effects of mean age, percentage of females, country, and tools of measurement were evaluated separately in univariate models. We found a significant moderating effect of the measurements of psychological inflexibility [*F*_(1, 12)_ = 5.82, *p* < 0.05]. It indicated a significantly larger pooled effect size estimate for AAQ-II (*r* = 0.69, 95% CI = 0.56, 0.83, *p* < 0.001) than that for PIPS (*r* = 0.49, 95% CI = 0.35, 0.64, *p* < 0.001). No significant moderating effects were found for the percentage of females, mean age, and country.

There was no publication bias in the Egger tests (*p* > 0.1) and fail-safe N analysis (*N* = 6,057) on the relationship between psychological inflexibility and anxiety, and further sensitivity analysis showed that the results were robust.

Only one study examined the relationship between psychological flexibility and anxiety, *r* = −0.57.

#### Psychological inflexibility and depression

**Figure 5:**
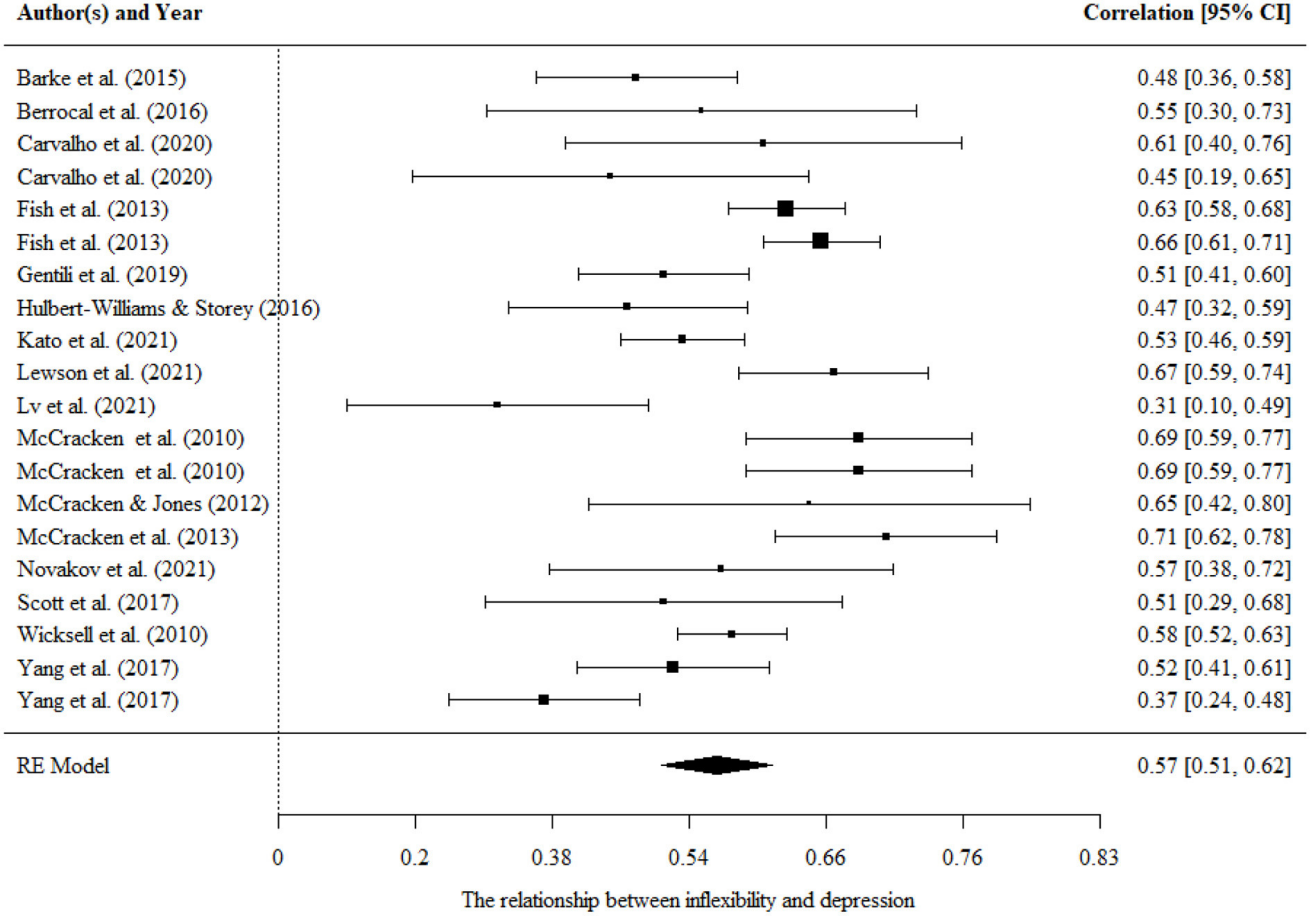
Forest plot for the relationship between psychological inflexibility and depression.

There were twenty correlations in sixteen studies that examined the relationship between psychological inflexibility and depression. It showed a large pooled effect size estimate (*r* = 0.57, 95% CI = 0.51, 0.62, *p* < 0.001). The results were presented in a forest plot in Figure 5.

There were significant variances within level 3. Moderating effects of mean age, percentage of females, country, the tools of measurement were evaluated separately in univariate models. We found a significantly moderating effect of the country [*F*_(1, 18)_ = 5.78, *p* < 0.05]. It indicated a significant larger pooled effect size estimate for the western country (*r* = 0.68, 95% CI = 0.61, 0.76, *p* < 0.001) than that for the eastern country (Δ*r* = −0.20, 95% CI = −0.37, −0.02, *p* < 0.05). No significant moderating effects were found for the percentage of females, mean age, and measurements.

There was no publication bias in the Egger tests (*p* > 0.1) and fail-safe N analysis (*N* = 10,769) on the relationship between psychological inflexibility and depression, and further sensitivity analysis showed that the result was robust.

No study examined the relationship between psychological flexibility and depression.

#### Psychological inflexibility/flexibility and quality of life

**Figure 6:**
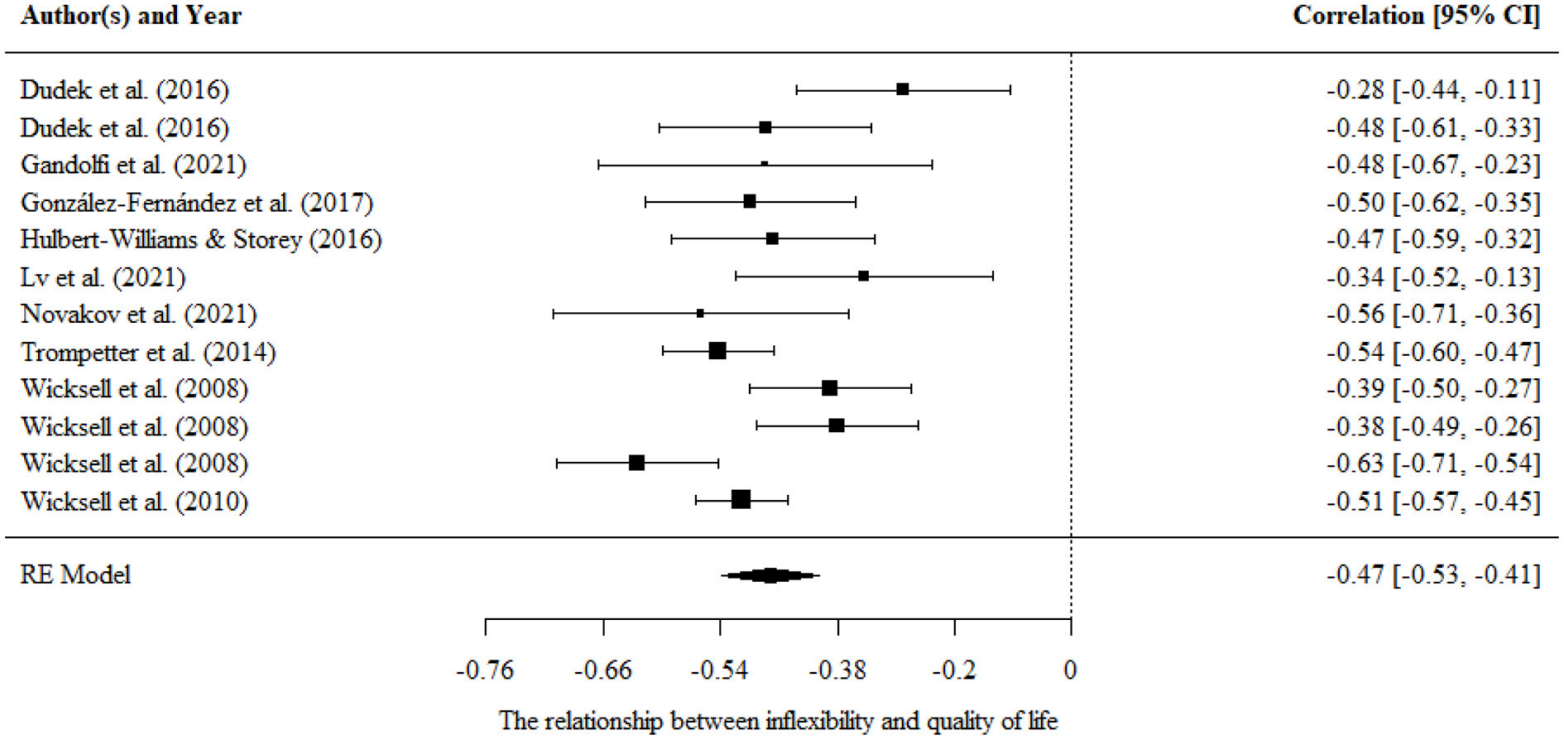
Forest plot for the relationship between psychological inflexibility and quality of life.

Aggregating across 12 correlations in 9 studies that examined the relationship between psychological inflexibility and quality of life, the results indicated a nearly large pooled effect size estimate (*r* = −0.47, 95% CI = −0.53, −0.41, *p* < 0.001). The results were presented in a forest plot in Figure 6.

There were significant variances within level 2. Moderating effects of mean age, percentage of females, country, and tools of measurement were evaluated separately in univariate models. However, no significant moderating effects were found.

There was no publication bias in Egger tests (*p* > 0.1) and fail-safe N analysis (*N* = 2,114) on the relationship between psychological inflexibility and quality of life, and further sensitivity analysis showed that the result was robust.

Only one study examined the relationship between psychological flexibility and quality of life, *r* = 0.37.

#### Psychological inflexibility and risk behaviors

Two studies examined the relationship between psychological inflexibility and risk behaviors (i.e., opioid use). It showed a non-significant pooled effect size estimate (*r* = 0.41, 95% CI = −0.18, 0.77, *p* > 0.1; *I*^2^ = 96.3%; *Q* = 27.1, *df* = 1, *p* < 0.001).

## Discussion

To date, no meta-analysis has summarized the relationship of psychological flexibility / inflexibility with these outcome variables in people with chronic pain for patients and clinical workers. A meta-analysis of this topic is important to better understand these relationships. Many studies have shown that ACT can improve the lives and reduce the symptoms of chronic pain patients by increasing their psychological flexibility or reducing psychological inflexibility. The elucidation of these relationships may provide possible explanatory mechanisms as to why ACT works in patients with chronic pain. The current study provided the first truly comprehensive perspective on the relationship between psychological flexibility/inflexibility and pain intensity/severity, quality of life, psychological distress, functioning, and risk behavior, and provided a conceptual and empirical basis for future work.

The present meta-analysis indicates a significant medium negative association between psychological flexibility and pain intensity or functional impairment. The present study also indicates a significant small to medium association between psychological inflexibility and pain intensity. There is a nearly large association between psychological inflexibility and functional impairment or the quality of life, and a large association between psychological inflexibility and anxiety/depression. Due to the limited number of included studies, the relationship between risk behavior and psychological inflexibility may not be significant. It is important to note that the relationship between psychological inflexibility and risk behavior was insignificant, and more future research is needed. There were not enough studies to aggregate the effect sizes of the relationship between psychological flexibility and anxiety, depression, quality of life, and risk behaviors. Previous research has suggested that psychological flexibility and inflexibility might represent distinct (yet related) processes and constructs (Rolffs et al., 2018; Daks and Rogge, 2020). Therefore, more research is needed to distinguish the two constructs.

This study is a research meta-analysis of correlational relationships. Therefore, it raises questions about causal inference, particularly about the possibility of inverse direction. However, pain catastrophizing, one of the strongest psychological predictors of pain outcomes (Schutze et al., 2018), can be explained by some essential key elements, such as repetitive negative thinking and rumination (Petrini and Arendt-Nielsen, 2020) which are some kind of overlapping with cognitive fusion (one of the core components of psychological flexibility) (McCracken et al., 2014). Besides, psychological flexibility and pain catastrophizing are considered as process mechanisms underlying the efficacy of ACT for pain patients (Trompetter et al., 2015). Furthermore, interventional studies in ACT provide a means to draw causal relationships because they aim to reduce chronic pain patients' psychological inflexibility and enhance their psychological flexibility (Hughes et al., 2017; Simpson et al., 2017; Trindade et al., 2021). These meta-analyses concluded that patients had significantly lower pain intensity, functioning, anxiety, depression, and psychological inflexibility after the ACT intervention than the control group. ACT is also one of the best evidence for reducing catastrophizing (Schutze et al., 2018). Nonetheless, these meta-analyses did not summarize the effects of ACT on the quality of life and risk behaviors in patients with chronic pain. Given the present study's findings, future intervention trials in the ACT may benefit from exploring the effects on quality of life and risk behaviors in chronic pain patients.

This study found that the type of country was a moderating variable in the relationship between psychological inflexibility and pain intensity or depression, significantly higher in chronic pain patients in western countries than in eastern countries. It is consistent with previous cross-sectional studies, which suggest that patients in Eastern countries may have a greater tolerance for pain (Thong et al., 2017; Yotnuengnit et al., 2022). This may be related to underlying cultural differences: stoicism is seen as a positive quality in Eastern cultures, whereas the inability to tolerate pain is seen as a sign of weakness (Narayan, 2010; Thong et al., 2017). We also found that the instruments of psychological inflexibility (i.e., AAQ-II, PIPS) moderate the relationship between psychological inflexibility and anxiety. The pooled effect sizes measured with AAQ-II were significantly larger than that with PIPS. This may be due to the construct and discriminant validity of AAQ-II. Some researchers highlighted questions surrounding the psychometric properties of the AAQ-II (Cherry et al., 2021; Garner and Golijani-Moghaddam, 2021), which suggested that the AAQ-II may measure psychological distress rather than psychological inflexibility. Thus, the correlation between psychological flexibility and anxiety, as measured by the AAQ-II, was higher. But this does not explain why the different instruments of psychological inflexibility had no impact on the relationship between psychological inflexibility and depression. In any case, it is necessary to develop alternative measures to more accurately capture the process of psychological inflexibility, which has been done by many authors (Francis et al., 2016; Rolffs et al., 2018). However, the validity of these instruments also deserves to be examined in future studies.

In the present study, no moderating variables were found that explained the heterogeneity of the relationship between psychological inflexibility and dysfunction. This may be due to the fact that the present study did not distinguish between psychological and physiological functioning, and included the both two distinct subcomponents in the heading of functioning. A recent study has specifically explored the relationship between the six subcomponents of psychological flexibility and functioning (Ding and Zheng, 2022). This study identified the measurement instrument of functioning as a possible factor contributing to heterogeneity (Ding and Zheng, 2022). Perhaps this study can give deeper insight into the relationship between psychological flexibility and functioning.

Given these associations, it seems that the way chronic pain patients relate to the difficult experiences they encounter may be implicated in their pain intensity, quality of life, functioning, and mental health symptomology. For instance, chronic pain patients who can focus on the present situation and move toward their goals may also experience improved professional quality of life and functioning, and lower levels of pain intensity or mental health symptomology. This may be due to the fact that psychological flexibility could provide less painful experiences and psychological distress in the pursuit of value-oriented behavior and reduce long-term distress and increase the ability to cope with chronic pain (Hayes et al., 2014; Goldbart et al., 2020). While for those who were trapped in their negative thinking or avoidance (i.e., psychological inflexibility), they may experience more anxiety, depression, dysfunction, and lower levels of quality of life which in turn may exacerbate the pain over time (Maathz et al., 2020; Rhodes et al., 2021). As such, targeting psychological inflexibility/flexibility may likely be valuable for chronic pain patients. As current literature highlights, ACT toward improving chronic pain patients' psychological flexibility seems to have desirable effectiveness in managing chronic pain (Hughes et al., 2017; Trindade et al., 2021). However, training in psychological flexibility should not be considered a panacea, and the responsibility for mental health, quality of life, or functioning should not be considered exclusively lying within the individual.

There were some limitations in the present study. First, a major weakness of this meta-analysis is that the studies' methodological quality was not rated. It was suggested that rating would be difficult due to the lack of clear methodological standards and relevant detail in the Methods sections of these studies (Levin et al., 2012; Low et al., 2020). This study excluded unpublished studies, providing a general approach to ensure methodological quality, but also raising the risk of publication bias affecting the results. A more comprehensive search of the published and unpublished literature may be useful for further research in this area. Second, the studies included were based on cross-sectional data, so the direction of causality remains unclear. Thus, the results need to be interpreted with caution. Third, we did not contact any study authors whose publications provide insufficient data about the correlation coefficient. It may have reduced the total number of studies included in analyses. Despite these limitations, the current study provided the first truly comprehensive perspective on the relationship between psychological flexibility/inflexibility and pain intensity/severity, quality of life, psychological distress, and functioning, providing a conceptual and empirical basis for future work.

Future research should distinguish psychological flexibility from inflexibility to better understand the psychological flexibility model in chronic pain. This meta-analysis suggests that the magnitude of effect sizes between psychological inflexibility/flexibility and functional impairment is different. Although recent research has shown that psychological flexibility and inflexibility are two different concepts (Rolffs et al., 2018; Daks and Rogge, 2020), no additional studies have shown differences in effect sizes between psychological inflexibility/flexibility and mental symptomology for chronic pain patients. In addition, if future research can combine psychological flexibility/inflexibility with the strong predictors of pain outcomes (such as pain catastrophizing), it will be more helpful to understand the mechanisms by which the psychological flexibility model works in patients with pain. Besides, it would also be beneficial to include more behavioral measures in studies for people with chronic pain. As the goal of ACT to enhance psychological flexibility is to help individuals pursue value-based goals, measures to assess behavior or behavioral change would be more useful to evaluate the psychological flexibility model in chronic pain. In addition, more research on long-term outcomes would be worthwhile. Although some evidence suggested psychological flexibility plays an important role in the development of pain and in long-term adaptation to pain (Linton and Shaw, 2011), the studies included were mostly cross-sectional. Also, data should be expanded beyond self-report data and collected from multiple informants to reduce reporting biases. Furthermore, it would be helpful to develop tools or use measures related to chronic pain. And, clinical workers should take note of efficient and cost-effective interventions in reducing chronic pain and functional impairment as well as improving mental health or quality of life for chronic pain patients.

## Data availability statement

The original contributions presented in the study are included in the article/supplementary material, further inquiries can be directed to the corresponding author.

## Author contributions

SF: conceptualization and project administration. SF and DD: methodology, data collection, data curation, formal analysis, visualization, writing–original draft, and writing–review and editing. Both authors contributed to the article and approved the submitted version.

## Funding

This study was supported by Philosophy and Social Science Planning Key Project of Anhui Province (Grant Number AHSKZ2020D37).

## Conflict of interest

The authors declare that the research was conducted in the absence of any commercial or financial relationships that could be construed as a potential conflict of interest.

## Publisher's note

All claims expressed in this article are solely those of the authors and do not necessarily represent those of their affiliated organizations, or those of the publisher, the editors and the reviewers. Any product that may be evaluated in this article, or claim that may be made by its manufacturer, is not guaranteed or endorsed by the publisher.
